# A multifunctional lipid nanoparticle for co-delivery of paclitaxel and curcumin for targeted delivery and enhanced cytotoxicity in multidrug resistant breast cancer cells

**DOI:** 10.18632/oncotarget.16153

**Published:** 2017-03-13

**Authors:** Jong-Suep Baek, Cheong-Weon Cho

**Affiliations:** ^1^ College of Pharmacy and Institute of Drug Research and Development, Chungnam National University, Yuseong-gu, Daejeon 34134, South Korea

**Keywords:** solid lipid nanoparticle, multidrug resistance, p-glycoprotein, targeting delivery system, folate-mediated

## Abstract

The objective of the work was to develop a multifunctional nanomedicine based on a folate-conjugated lipid nanoparticles loaded with paclitaxel and curcumin. The novel system combines therapeutic advantageous of efficient targeted delivery via folate and timed-release of curcumin and paclitaxel via 2-hydroxypropyl-ß-cyclodextrin, thereby overcoming multidrug resistance in breast cancer cells (MCF-7/ADR). The faster release of curcumin from the folate-conjugated curcumin and paclitaxel-loaded lipid nanoparticles enables sufficient p-glycoprotein inhibition, which allows increased cellular uptake and cytotoxicity of paclitaxel. In western blot assay, curcumin can efficiently inhibit the expression of p-glycoprotein, conformed the enhancement of cytotoxicity by paclitaxel. Furthermore, folate-conjugated curcumin and paclitaxel-loaded lipid nanoparticles exhibited increased uptake of paclitaxel and curcumin into MCF-7/ADR cells through the folate receptor-mediated internalization. Taken together, these results indicate that folate-conjugated curcumin and paclitaxel-loaded lipid nanoparticles enables the enhanced, folate-targeted delivery of multiple anticancer drugs by inhibiting the multi-drug resistance efficiently, which may also serve as a useful nano-system for co-delivery of other anticancer drugs.

## INTRODUCTION

Breast cancer is one of the most common cancers for women and still a major reason of death. Among breast cancer cell lines, MCF-7/ADR cells are known as multi-drug resistance (MDR) expressing cells. The development of MDR is one of the major challenges leading to the failure of several conventional chemotherapies [1, 2]. The increased efflux of hydrophobic drugs from the cell by ATP-binding cassette (ABC) transporter trans-membrane proteins such as p-glycoprotein (p-gp, also known as MDR1) results in a prevention of drug accumulation, thus reducing the sensitivity of cancer cells to anticancer drugs [3]. An attractive approach to overcome MDR expressing cancer cells is to co-delivery p-gp inhibitor along with the anticancer drug, thereby achieving enhanced accumulation of the anticancer drug and therapeutic efficacy [4–8].

Paclitaxel, the microtubule-stabilizing agents, has been used as an effective chemotherapeutic agent for various solid tumors such as ovarian, breast, and lung cancers [9]. Although paclitaxel is the first choice for the treatment of breast cancer, its use is limited due to the limited uptake of the drug by p-gp. Curcumin, a polyphenol extracted from the rhizome of the plant *Curcuma longa* Linn (turmeric). It has a variety of pharmacologic effects including anti-amyloid, anti-oxidant, anti-inflammatory, anti-bacterial, and anti-spasmodic activity without any major side effects [10–12]. Specially, several groups have reported that curcumin could be used as a chemotherapeutic agent [13, 14]. Furthermore, some studies demonstrated the curcumin independently exhibited p-gp inhibitory activity by down-regulation of the PI3K/Akt and NF-kB pathways [15, 16]. Therefore, curcumin could be used as an effective p-gp inhibitor on MDR-expressing cancer cells by co-administration with an anticancer drug to maximize the cytotoxicity of the anticancer drug. However, the low solubility and poor stability of curcumin in physiological environment result in its poor bioavailability and therapeutic effect [17].

On the other hand, conventional co-delivery systems showed a few disadvantages such as unsuitable release profiles, use of conventional p-gp inhibitors which have toxicity to normal cells and non-targeted property. Hence, ideal delivery systems for combination should have targeting property and optimal release profile. Namely, the chance of exposure of anticancer agent and p-gp inhibitor to normal cells or tissues should be minimized and localized in a specific targeted cells or tissues. Furthermore, this system should use non-toxic p-gp inhibitor such as curcumin instead of conventional p-gp inhibitor such as verapamil and dronedarone that have toxicity [18]. However, conventional paclitaxel and curcumin nanosystems have been reported to delivery two drugs in similar release pattern [19]. The similar release rate of paclitaxel and curcumin would be an additional issue due to insufficient release of curcumin in advance of release of paclitaxel. In order to address the issues, it is hypothesized that curcumin and paclitaxel can be timed-released from our novel system, providing a controlled and sustained drug release, along with maximized anticancer efficacy. Considering the modulation of drug release to inhibit p-gp first, our group developed inclusion complex-loaded solid lipid nanoparticles (SLNs) that enabled to release p-gp inhibitor faster than anticancer drug [20]. SLNs have been used in wide range of properties owing to low toxicity and good potential in cosmetic, food and pharmaceutical fields [21–23].

**Figure 1:**
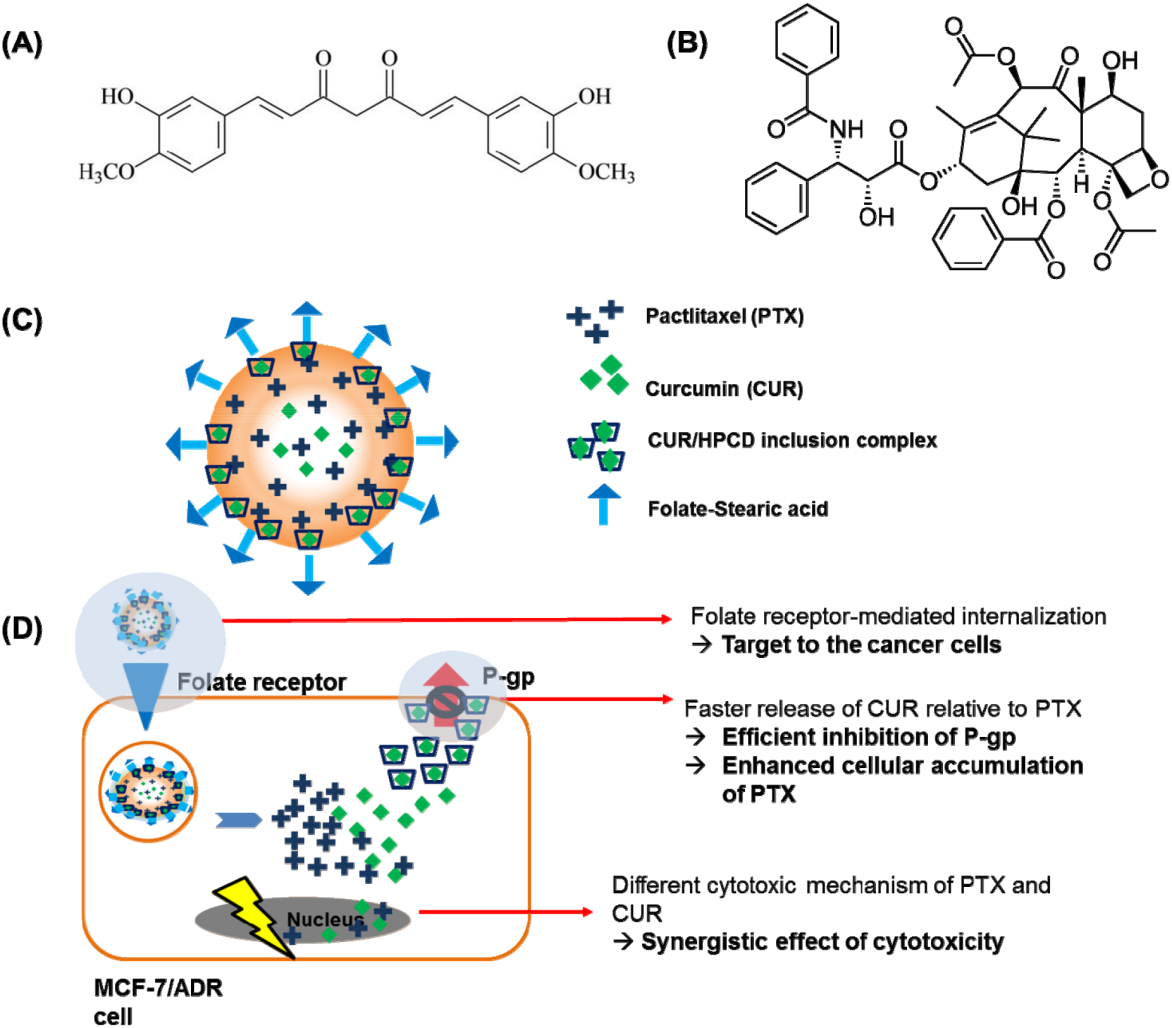
The structure of curcumin (A) and paclitaxel (B). **(C)**, Schematic illustration of the folate conjugated paclitaxel and curcumin/HPCD co-loaded lipid nanoparticles (FPCHN-30); **(D)**, The folate moieties on the surface of nanoparticles allow for active uptake by over-expressed folate receptors on MCF-7/ADR cells and subsequently release curcumin and paclitaxel in sequential manner. Released curcumin is readily able to inhibit the expression of p-glycoprotein (p-gp) to enhance paclitaxel intracellular accumulation and to maximize its cytotoxicity. Meanwhile, curcumin also can exhibit cytotoxicity with its own mechanism.

Herein, we have encapsulated curcumin in 2-hydroxypropyl-β-cyclodextrin (HPCD) to improve its stability and water-solubility for faster release relative to the release of paclitaxel (Figure 1A). As a result, both the anticancer drug (paclitaxel) and the chemosensitizer (curcumin) were able to be efficiently delivered to the target breast cancer cells at the same time. It is highlighted that curcumin from curcumin/HPCD inclusion complex can be released faster, leading to the sufficient p-gp inhibition for enhanced intracellular accumulation of paclitaxel against MCF-7/ADR cells (Figure 1B). Moreover, the ability to delivery huge amount of anticancer drug to the target cancer cells can enable to reduce the systemic side effects related to the most of anticancer drugs [24, 25]. In order to achieve the targeting ability, we have further conjugated folate to stearic acid as a targeting moiety because folate binds to the folate receptors that are widely over-expressed on surfaces of specific cancer cells such as MCF-7/ADR cells.

## RESULTS

### Preparation and characterization of various NPs

The nanoparticles were fabricated by emulsification and the solvent evaporation method. The physicochemical properties including particle size, polydispersity index (PDI), zeta potential and encapsulation efficiency (EE) were listed in Table 1. PCN was shown to be insignificantly different as compared to that of PN. The particle size of PCN was 149.6 ± 6.9 nm, after modified with FA, the size slightly increased to 161.5 ± 7.1 nm. On the other hand, the particle size in FPCHN series showed the increasing tendency according to the amount of HPCD. As for zeta potential, all of NPs exhibited similar values.

**Table 1:** The physicochemical characteristics of different nanoparticles (n=3, mean ± SD). *p < 0.05 compared to FPCN

Formulation	Particle size (nm)	Polydispersity index	Zeta potential (mV)	Encapsulation efficiency (%)	
				Paclitaxel	Curcumin
PN	140.3 ± 7.9	0.156 ± 0.007	−27.5 ± 6.1	87.5 ± 3.5	-
PCN	149.6 ± 6.9	0.165 ± 0.012	−26.8 ± 5.8	81.3 ± 4.8	83.6 ± 3.9
FPCN	161.5 ± 7.1	0.221 ± 0.031	−22.3 ± 2.7	79.1 ± 4.2	85.7 ± 5.2
FPCHN-10	169.1 ± 9.3	0.226 ± 0.033	−21.1 ± 4.3	77.3 ± 5.1	79.5 ± 6.1
FPCHN-30	175.5 ± 6.2	0.246 ± 0.016	−26.8 ± 4.4	78.7 ± 5.9	76.2 ± 4.6
FPCHN-50	215.9 ± 8.3	0.357 ± 0.025	−23.5 ± 5.2	70.1 ± 8.6	51.1 ± 10.7*

EE (%) of paclitaxel and curcumin in all of NPs observed to be above 75% except FPCHN-50. Interestingly, co-encapsulation of curcumin with paclitaxel in PCN resulted in slightly low EE of paclitaxel (81.3 ± 4.8%) as compared to that of PN (87.5 ± 3.5%). However, FA-conjugation did not impair EE of paclitaxel and curcumin. It was noted that the amount of HPCD used resulted in decreasing EE of curcumin, while EE of paclitaxel was not significantly changed. Specially, the EE of curcumin in FPCHN-50 was significantly lower than that of FPCN, while that of FPCHN-10 and -30 was insignificant different compare to that of FPCN.

Surface modification of PCN with folate was confirmed using XPS. XPS result of PCN did not show nitrogen on the surface of NPs (Table 2), while FPCN showed nitrogen on the surface. FPCN exhibited 9.5% atomic concentration of nitrogen on the surface at 399.7 ev, indicating successful conjugation of SA-FA on the surface of FPCNs.

**Table 2:** BE position and atomic concentration of C, N and O in FPCN and CPS

Formulation	Name	BE (ev) Position	Atomic concentration (%)
FPCHN-30	C	284.9	78.5
	N	399.7	9.5
	O	532.1	12.0
PCN	C	285.2	79.5
	N	-	-
	O	533.0	20.5

### *In vitro* release

**Figure 2:**
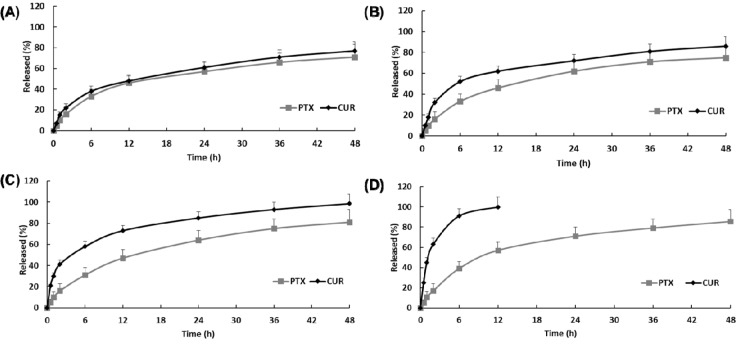
*In vitro* release of curcumin (CUR) and paclitaxel (PTX) in 0.1 % (w/v) Tween 80 solution (n=3, mean ± SD) **(A)**, FPCN; **(B)**, FPCHN-10; **(C)**, FPCHN-30; **(D)**, FPCHN-50.

*In vitro* drug release of paclitaxel and curcumin from FPCN and FPCHN was conducted in PBS with 0.1% (w/v) tween 80. In order to achieve optimal sequential release profile of curcumin and paclitaxel, different amount of HPCD (0, 10, 30 or 50 mg) was introduced to form inclusion complex with curcumin. As shown in Figure 2, FPCN exhibited similar release profile of paclitaxel and curcumin over 48 h due to similar hydrophobicity of drugs. On the other hand, the release profile of FPCHN revealed a faster release of curcumin than that of paclitaxel at all the time points. Notably, release rate of curcumin was observed to be faster with the used amount of HPCD. The initial burst release of curcumin from FPCHN-10, 30 and 50 at 2 h was 32.3 ± 4.7%, 41.3 ± 6.8% and 63.8 ± 7.1%, respectively. In particular, FPCHN-50 exhibited huge burst release of curcumin among the FPCHNs and all of curcumin was fully released in 12 h. Interestingly, the release rate of paclitaxel was statistically insignificant different among the all FPCHNs. It was emphasized that the release of curcumin from FPCHNs exhibited faster at all times in comparison with that of paclitaxel and can be adjusted with different amount of HPCD. For further studies, the FPCHN-30 was chosen in consideration of the particle size, polydispersity index, encapsulation efficiency and release profile of curcumin and paclitaxel.

### Cellular uptake

**Figure 3:**
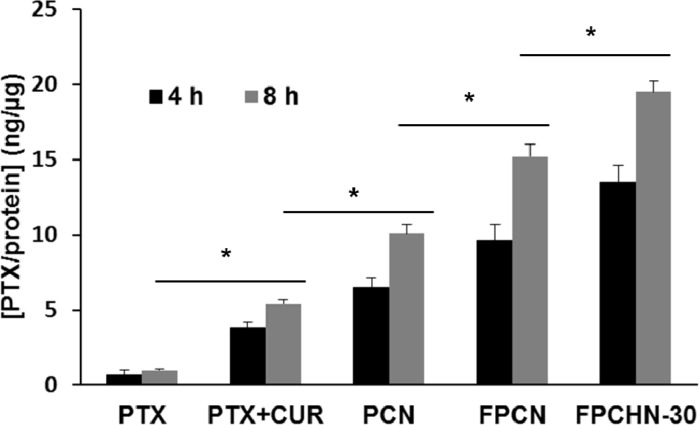
Cellular uptake of paclitaxel (PTX) (5 nM) from different formulations at different time points (4 and 8 h) in MCF-7/ADR cells (n=3, mean ± SD)

The quantitative cellular uptake of paclitaxel from the different formulations was presented in Figure 3. From the results, the co-treatment of paclitaxel and curcumin exhibited increased uptake of paclitaxel compared to that of paclitaxel alone. In addition, encapsulation of both drugs in nanoparticles allowed for enhanced uptake of paclitaxel compared to free paclitaxel and curcumin. Furthermore, FA-conjugation allowed for further enhancement of cellular uptake of paclitaxel by receptor-mediated uptake. Figure 3 shows that the uptake of paclitaxel of free paclitaxel+curcumin, PCN, FPCN and FPCHN-30 were higher than that of free paclitaxel. Except free paclitaxel, all of formulations exhibited time-dependent uptake of paclitaxel in MCF-7/ADR cells. At 8 h, co-treatment of curcumin and paclitaxel (free paclitaxel+curcumin) showed significant higher uptake of paclitaxel (5.1 ± 0.3 ng/μg) compared to that of free paclitaxel (1.1 ± 0.1 ng/μg). Further enhanced cellular uptake of paclitaxel (10.2 ± 0.8 ng/μg) was achieved by encapsulation of drugs in nanoparticles (PCN). Folate-conjugation of PCN (FPCN) also exhibited significant increment of internalized paclitaxel (14.9 ± 1.3 ng/μg) compared to that of non-conjugated PCN. Notably, FPCHN-30 exhibited the highest cellular paclitaxel uptake (19.1 ± 1.1 ng/μg) among the tested formulations and 1.44 times higher than that of FPCN.

**Figure 4:**
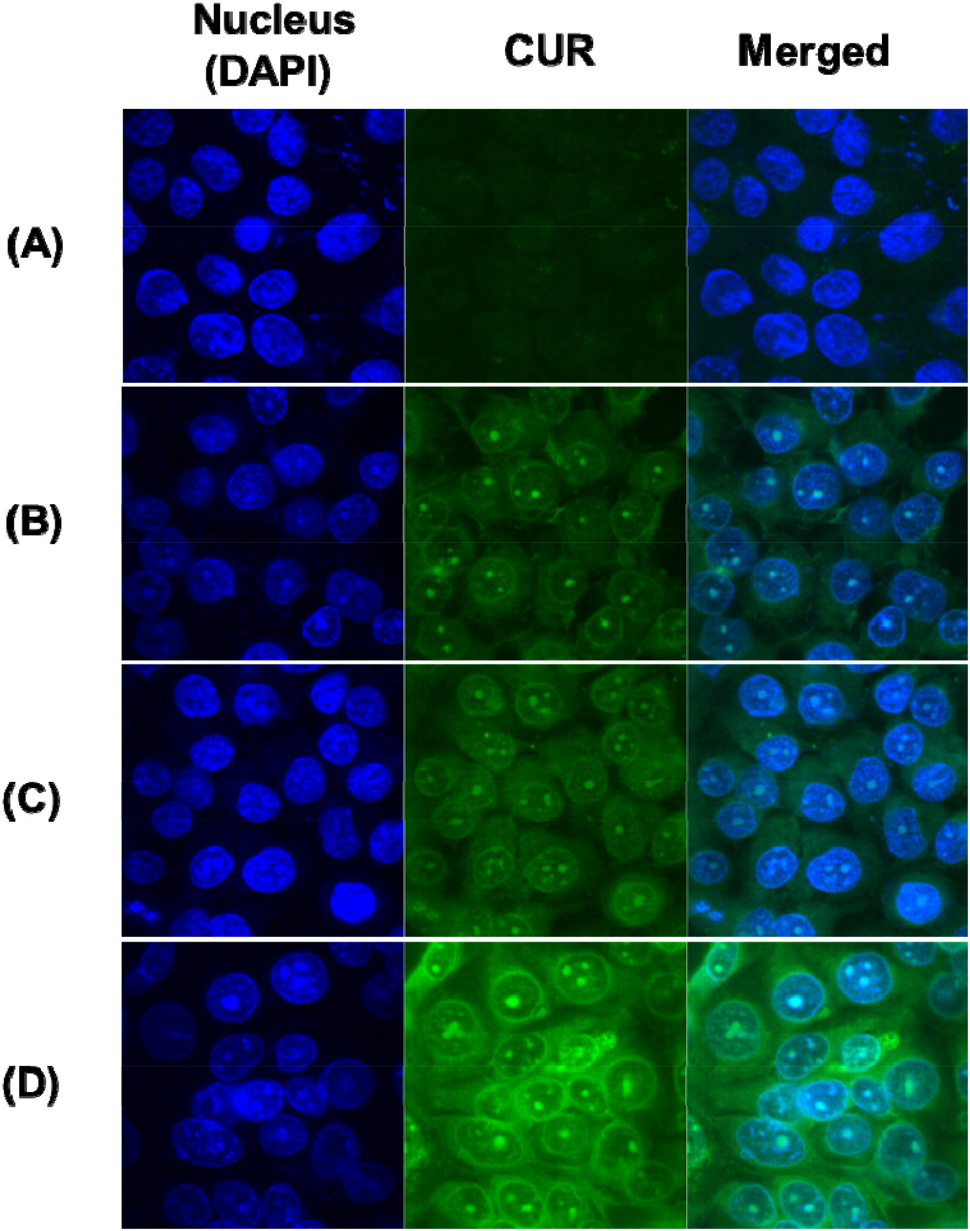
Cellular uptake of curcumin (CUR) (2.5 nM) at 4 h in MCF-7/ADR cells **(A)**, free CUR; **(B)**, PCN; **(C)**, FPCN; **(D)**, FPCHN-30.

In order to further investigate the uptake of curcumin in MCF-7/ADR cells, the fluorescence of curcumin was measured by confocal microscopy. This evaluation has been reported as the indirect method of nanocarrier penetration into the cells [26]. As shown in Figure 4, all of NPs allowed for higher uptake of curcumin into the cells than free curcumin alone. Similar to the uptake of paclitaxel, the uptake of curcumin was enhanced by nano-encapsulation, folate-conjugation, and modification of release of curcumin. As such, PCN exhibited more internalization of curcumin than free curcumin. In addition, additional internalization of curcumin was achieved by active-targeting of folate-conjugation. Specially, FPCHN-30 was able to be more internalized than FPCN.

**Figure 5:**
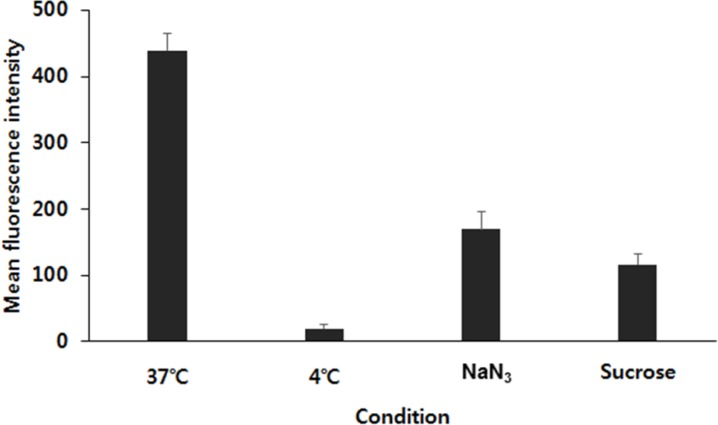
The mean fluorescence intensity of curcumin in FPCHN-30 under different incubate conditions in terms of different temperatures (37°C or 4°C) and co-treatment of 0.07 M of NaN3 or 0.45 M of sucrose for 4 h (n=3, mean ± SD)

Flow cytometry analysis was carried out to figure out the cellular uptake mechanism of FPCHN-30 on MCF-7/ADR cells. Firstly, an effect of temperature on cellular uptake was tested by incubating the cells with FPCHN-30 at 4 or 37°C for 4 h (Figure 5). The uptake of curcumin in FPCHN-30 decreased to 4.6% at 4°C as compared to the normal condition (37°C). Next, the endocytosis inhibitors, sodium azide (NaN_3_) and sucrose, were co-treated with FPCHN-30 in MCF-7/ADR cells, respectively. The introduction of NaN_3_ and sucrose reduced the mean intensity of curcumin to 38.7 and 26.5%, respectively.

### Cytotoxicity

**Figure 6:**
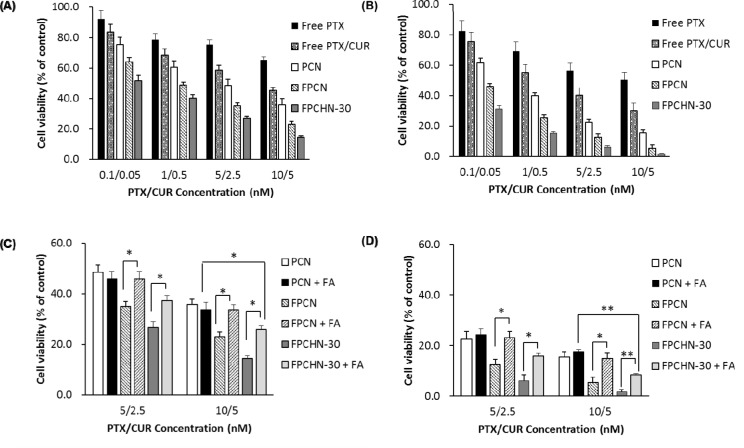
Cytotoxicity studies of different formulations for (A) 24 h and (B) 48 h in MCF-7/ADR cells Cytotoxicity of PCN, FPCN, and FPCHN-30 with or without FA for **(C)** 24 h, and **(D)** 48 h in MCF-7/ADR cells (n=3, mean ± SD). *p < 0.05; **p < 0.01.

The cytotoxicity of the different formulations against MCF-7/ADR cells was determined by the MTT assay for 24 and 48 h. As seen in Figure 6A and 6B, co-treatment of curcumin and paclitaxel (free paclitaxel/curcumin) induced cell viability compare to that of free paclitaxel at all tested concentrations. Similarly, to uptake results, nano-encapsulation and folate-conjugation allowed for increment of cytotoxicity. Notably, it was observed that FPCHN-30 exhibited the highest cytotoxicity among tested formulations on MCF-7/ADR cells. All of NPs exhibited time-dependent cytotoxicity.

Subsequently, the effect of folate targeting on the cytotoxic potential of the PCN, FPCN and FPCHN-30 on the MCF-7/ADR cells was evaluated (Figure 6C and 6D). As expected, the results showed that the cell viability of folate-conjugated formulations including FPCN and FPCHN-30 was recovered when FA was co-treated. Nevertheless, it was highlighted that FPCHN-30 with FA exhibited significantly induced cell viability in the tested formulations (*p* < 0.01).

### Effect of folate targeting and sequential release of curcumin and paclitaxel on protein levels of p-gp

**Figure 7:**
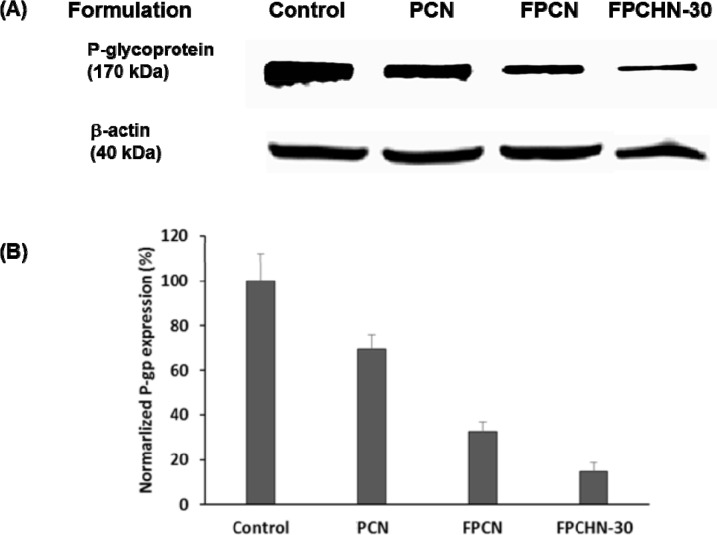
The expression of (A) p-gp, and β-actin and (B) normalized p-gp integrity after treatment of PCN, FPCN, and FPCHN-30 for 4 h in MCF-7/ADR cells

Western blot assays were conducted to confirm the inhibition of p-gp expression in MCF-7/ADR cells treated with different formulations. As shown in Figure 7, co-treatment of curcumin with paclitaxel inhibited p-gp expression compared to that of untreated cells. Additional inhibition of p-gp expression was achieved by folate-conjugation. Notably, FPCHN-30 was observed to maximize inhibition of p-gp expression in the formulations tested. Equal protein loading was verified by the integrity of β-actin.

**Figure 8:**
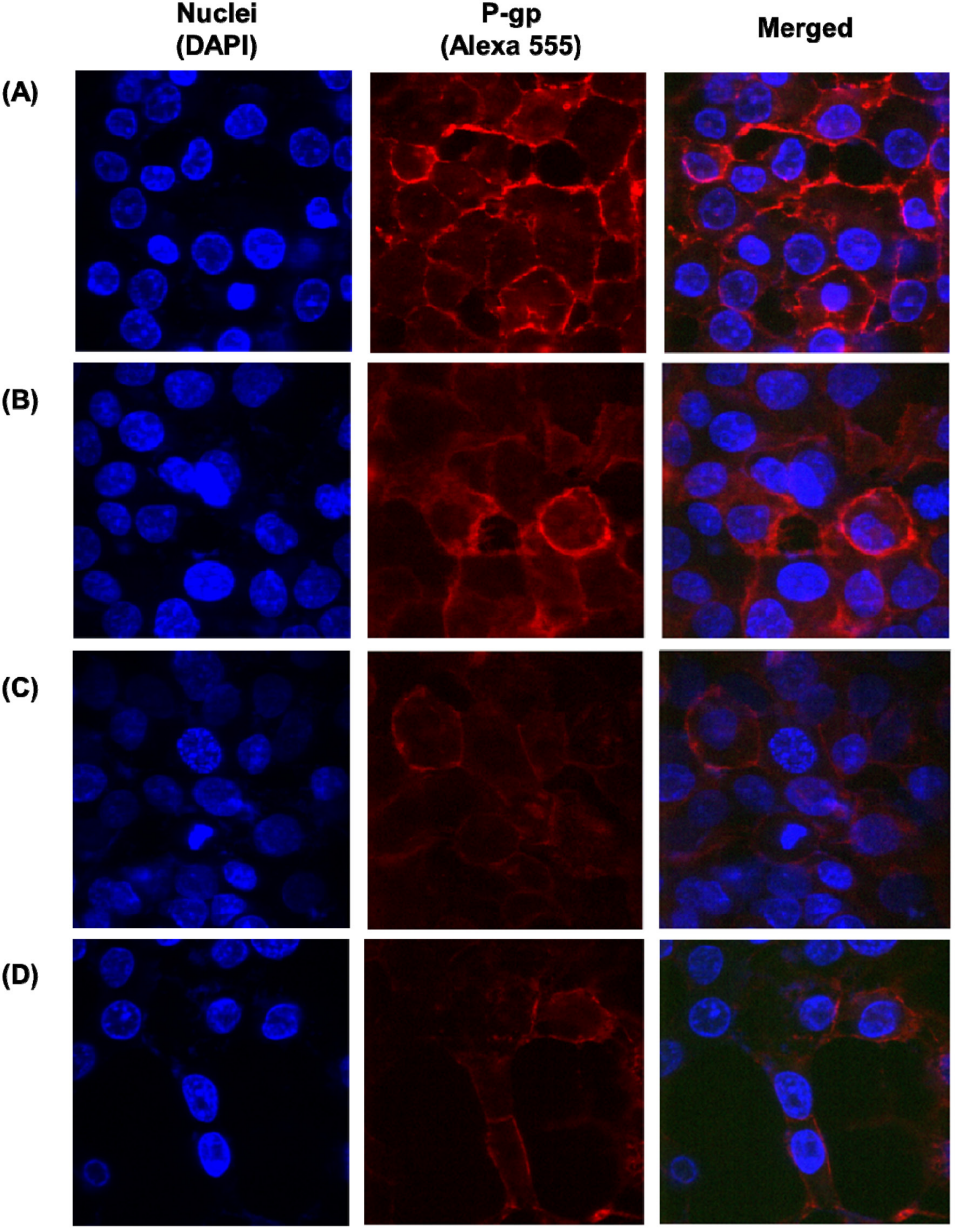
The expression of p-gp on the surface of MCF-7/ADR cells **(A)** no treatment and after treatment of **(B)** PCN, **(C)** FPCN, and **(D)** FPCHN-30 for 4 h. The p-gp was stained by 1st MDR antibody followed by 2^nd^ Alexa 555 antibody.

Immunofluorescence staining was further conducted to visualize the expression of p-gp (Figure 8). In order to investigate the effect of different formulations, the chosen formulations were incubated with MCF-7/ADR cells for 4 h. Similarly, to the result of western blot, the co-treatment of paclitaxel and curcumin showed p-gp inhibitory effect compared to that of untreated cells. In addition, FA targeting further allowed for reduced p-gp expression. Specially, FPCHN-30 suppressed p-gp the most in the formulations tested.

## DISCUSSION

MDR is a major limitation for a treatment of cancer because most of cancer-induced deaths are attributed to metastases that are resistant to anticancer drugs [3, 27]. A first choice of the treatment of drug-resistant tumors is a combination therapy of anticancer drugs. The therapeutic effect of such combination therapy, however, is often insufficient due to the different pharmacokinetics of the drugs [28] and uncontrolled release for overcoming MDR [29]. A promising approach for addressing the issues is the use of nanocarrier that concurrently encapsulates and releases multiple drugs in controlled manner, while providing a targeting ability to the cancer cells.

In this study, a novel FA-conjugated targeting and sequential releasing of multiple drugs (i.e. paclitaxel and curcumin) loaded lipid nanoparticles (FPCHN) was developed for the smart approach on the efficient treatment in MDR expressing MCF-7/ADR cells. Paclitaxel is known as the first-line for the treatment of breast cancer. Recently, curcumin has been known as p-gp inhibitor as well as anticancer drug. Hence, it was hypothesized that first release of curcumin could inhibit p-gp and increase the cellular uptake of paclitaxel. A few studies have reported to overcome p-gp and enhance cytotoxicity by co-administration of a p-gp inhibitor with anticancer drug [20, 30]. Besides, both of drugs could show synergistic effect because curcumin and paclitaxel have different cytotoxic mechanisms. It is known that paclitaxel induces the apoptosis of cancer cells by stopping microtubules disassembly, thereby inhibiting cell division [31]. On the other hand, curcumin is known to block the transcriptional factor nuclear factor (NF)-κB that is a major inhibitor of cell proliferation, apoptosis and resistance of cells [15, 16]. Hence, co-treatment of curcumin and paclitaxel could enhance cytotoxicity against cancer cells compare to conventional single chemotherapy. In order to maximize the synergistic and therapeutic effects and minimize drug-drug interaction of paclitaxel and curcumin, sequential release of curcumin and paclitaxel was achieved by the addition of HPCD. The FPCHN was successfully conjugated with FA for the targeting to the folate receptor-expressing MCF-7/ADR cells. The addition of SA-FA to the lipid phase could increase the amount of lipid in the organic phase and results in increased viscosity of the in particle size [30].

The particle size range of all NPs was shown to be less than 200 nm except FPCHN-50. Such physical characteristics of all NPs in the range of 10-200 nm are likely advantageous to the NPs in exploiting the enhanced permeation and retention (EPR) effect against solid tumors through the leaky vasculature [32, 33]. The use of HPCD (50 mg) caused negative effect on FPCHN in terms of size, polydispersity index, and encapsulation efficiency while less amount of HPCD (10 and 30 mg) did not impair physicochemical properties of formulations.

The hydrophobicity can enhance EE and decrease release rate of drug in lipid nanoparticles. Hydrophobic drug can strongly interact with hydrophobic lipid matrix, thus allowing high EE and sustained release. Meanwhile, it was reported that the increased particle size of FPCN results in increasing the distance between the core and the surface [30]. This is why the release of both drugs loaded in FPCN was less at all times in comparison with that of PCN. Specially, FPCHN showed the controlled release of both drugs, and release of curcumin was faster than release of paclitaxel because curcumin/HPCD may be located near surface of NPs due to lower lipophilicity of curcumin in comparison with that of paclitaxel [20]. For release of paclitaxel, insignificant difference was observed in tested formulations. On the other hand, the release profile of curcumin for FPCNHs was accelerated according to the amount of HPCD. It was noted that the release of curcumin from FPCHNs was faster than that of paclitaxel at all-time points, while FPCN exhibited similar release of curcumin and paclitaxel. It was reported that the release of drugs from lipid nanoparticles can be influenced by the nature of the lipid matrix, surfactant concentration and production parameters as well as solubility of the drug in lipid matrix and partition coefficient [34–37]. From this perspective, similar lipophilicity of curcumin (log P: 3.29) and paclitaxel (log P: 3.96) could be reason for similar release of both drugs from FPCN. On the other hand, faster release of curcumin from FPCHNs is attributed to the hydrophilic nature of surface of HPCD, which facilitates complete solubilization of curcumin in the release medium [20]. The hydrophilic curcumin/HPCD complex was localized near surface of lipid nanoparticles due to weak interaction between lipid and inclusion complex, which providing faster release of curcumin/HPCD from lipid nanoparticle [38]. Among tested FPCHNs, FPCHN-30 was chosen after taking into consideration of EE and release profile. FPCHN-30 exhibited about 40% of burst release, followed by sustained release up to 48 h as well as similar encapsulation efficiency of curcumin and paclitaxel as compared to that of FPCN. The faster release of curcumin provided the opportunity to inhibit p-gp expression, whereby followed release of paclitaxel could be easily taken up by MCF-7/ADR cells.

The cellular uptake of different formulations was evaluated in MCF-7/ADR cells. The free paclitaxel showed poor cellular uptake of paclitaxel due to the p-gp efflux, while all of NPs prepared were easily taken up by the cells [39, 40]. Co-treatment of curcumin with paclitaxel enabled for further enhanced internalization of paclitaxel as compared to that of free paclitaxel, which indicating the p-gp inhibition of curcumin [41, 42]. In addition, FA-conjugated NPs exhibited further enhancement of internalization of paclitaxel. The active targeting interaction between the FA and the FA-receptor overexpressed on membrane of MCF-7/ADR cells allows FPCN and FPCHN-30 to be easily taken up by the cells. As expected, sequential release of curcumin and paclitaxel from FPCHN-30 exhibited the highest paclitaxel uptake among tested formulations. All of cellular uptake results obtained were well correlated with cytotoxicity results.

Next, flow cytometry analysis was conducted to figure out the cellular uptake mechanism for the FPCHN-30. Firstly, the effect of temperature on cellular uptake was studies by incubating the cells with FPCHN-30 at 37°C or 4°C for 4 h. As seen in Figure 5, the uptake of FPCHN-30 was significantly reduced by 95% at 4°C due to a frozen state of cell membranes. So, particles were not able to be internalized via interaction between particles and cell membranes, which results in a significant decrease of their uptake. Sodium azide (NaN_3_) is widely used as endocytosis inhibitor agent which is known to disturb the production of ATP in cells and block the endocytotic pathway [43]. In this study, mean fluorescence intensity of curcumin from FPCHN-30 was reduced by about 60%. Sucrose is known to disrupt the formation of clathrin-coated vesicles and used as an endocytosis inhibitor [44]. In this study, pretreatment with sucrose significantly reduced the uptake of FPCHN-30 by about 70%. These results suggested that internalization of FPCHN-30 depended on a temperature-, energy- and clathrin-dependent pathway.

In order to confirm the enhanced cellular uptake of paclitaxel and curcumin, cell cytotoxicity was measured by MTT assay. All the formulations exhibited higher cytotoxicity as compared to that of free paclitaxel and curcumin. In addition, folate conjugation onto surface of NPs allowed for further cytotoxicity. It was highlighted that FPCHN-30 exhibited still higher cytotoxicity compared to that of FPCN when the NPs were co-incubated with folate. FPCHN-30, rate-controlling and active-targeting delivery system, exhibited the highest cytotoxicity as well as cellular uptake on MCF-7/ADR cells.

Finally, the p-gp inhibition efficiency of FPCHN-30 was evaluated by western blot assay and immunofluorescence staining. From the results obtained, FPCHN-30 exhibited maximum inhibition of p-gp among tested group. Similarly, to the results of cell studies, co-treatment of curcumin exhibited depressed p-gp expression as compared to that of paclitaxel alone. Specially, sequential release of curcumin and paclitaxel from FPCHN-30 was observed to have better inhibition of p-gp, strongly indicating the importance of release profile of both drugs and active targeting to achieve the enhanced therapeutic efficiency. The faster release of curcumin was able to inhibit p-gp efficiently so that paclitaxel could be easily internalized in MCF-7/ADR cells.

On the basis of our experimental studies, FPCHN-30 could be used as a promising therapeutic delivery system for MDR-expressing breast cancer cells by sequential release of p-gp inhibitor and anticancer drug, and active targeting to FA receptors (Figure 1A and 1B).

## CONCLUSIONS

From this study, we presented a promising approach to improve the cellular uptake of paclitaxel and curcumin into MCF-7/ADR cells that express folate receptors and p-gp. Our novel system exhibited a significant enhanced cytotoxicity and cellular uptake from the combined effects of optimized sequential release of multiple drugs and folate receptor-mediated internalization by manipulating hydrophilicities and conjugation of folate with lipid. Since targeting ability and controlled release of curcumin and paclitaxel are achievable with FPCHN-30, this nanocarrier would have great potential to inhibit MDR-expressing tumor cells.

## MATERIALS AND METHODS

### Materials

Paclitaxel was provided by Samyang Genex (Daejeon, Korea). Stearic acid was purchased from Daejung Chemical (Cheongwon, Korea). Poloxamer 188 was obtained from BASF (Ludwigshafen, Germany) and lecithin was acquired from Junsei (Tokyo, Japan). Curcumin, HPCD, folate, DAPI, Nile Red and mannitol were purchased from Sigma-Aldrich (Steinheim, Switzerland). All other chemicals and reagents used were of analytical grade.

### Preparation of surface-modified paclitaxel and curcumin loaded solid lipid nanoparticles

Paclitaxel and curcumin loaded lipid nanoparticles (PCN) were prepared using emulsification and the solvent evaporation method [45]. Briefly, paclitaxel (10 mg), curcumin (5 mg), glyceryl monostearate (GMS, 100mg) and TPGS (100 mg) were dissolved in 10 mL of chloroform to produce an oil phase. The oil phase was added to the aqueous phase containing 1.5% Tween 80 and homogenized (Ultra Turrax T25) for 3 min at 12,000 rpm. The obtained emulsion was sonicated for 5 min. In order to achieve optimal release profile of curcumin, curcumin/HPCD was synthesized with different amount of HPCD (10, 30 or 50 mg).

Folate-conjugated curcumin and paclitaxel loaded SLNs (FPCN) were prepared by adding 5 mg of conjugated stearic acid and folate (SA-FA) in the organic lipid phase using the same method described above. The SA-FA was synthesized using EDC (1-ethyl-3(3-dimethylaminopropyl)carbodiimide) coupling reaction [30, 39]. Presence of SA-FA on the surface was confirmed by XPS analysis.

### X-ray photon spectroscopy (XPS)

The presence of folate in SA-FA on the particle surface was confirmed using X-ray photoelectron spectroscopy (MultiLab 2000, Thermo). The lyophilized nanoparticle samples were applied on a clean aluminum substrate and kept under vacuum. The surface chemical composition on the nanoparticles was examined using a surface spectrophotometer. XPS analysis was conducted for PCN and FPCN.

### Analysis of drug loading and encapsulation efficiency

The lyophilized nanoparticles (10 mg) were solubilized with 1 mL of ethanol, heated at 80°C for 30 min and then cooled down at -20°C for 30 min. This solution was centrifuged at 10,000 g for 5 min to precipitate the undissolved solid lipid, filtered through a 0.22 μm filter and injected into the HPLC system. An Agilent 1100 liquid chromatography system with an autosampler and UV detector were used. The column used was a C_18_ column (4.0 × 250 mm, 5 μm particle size, Supelco^TM^; MetaChem, USA). The flow rate of the mobile phase was 1 mL/min and the detection wavelength of paclitaxel or curcumin was set to 227 nm or 425 nm, respectively. The mobile phase of paclitaxel or curcumin was a mixture of distilled water and acetonitrile (60:40, v/v) or distilled water and acetonitrile (30:70, v/v) adjusted pH 2.5 by formic acid, respectively. Drug loading and encapsulation efficiency (E.E) were calculated as follows:

Drug loading (%) = weight of the drug in particles/weight of the particles x 100.

E.E (%) = weight of the drug in particles/weight of the feeding drugs x 100.

### Measurements of particle size, polydispersity and zeta potential

The particle size and zeta potential analysis of different nanoparticles were performed by laser scattering analyzer (ELS-8000, Otasuka Electronics, Osaka, Japan). The lyophilized nanoparticles were dispersed in distilled water and sonicated to minimize the inter-particle interactions. The obscuration range was maintained between 20~50%. The instrument was set to measure the sample 100 times and the average volume mean diameter was obtained. The zeta potential of different nanoparticles was measured by Zetasizer Nano Z (Malven, UK). Data were collected as the average of 20 measurements.

### *In vitro* release study

*In vitro* release studies of different formulations were evaluated using a dialysis bag (molecular weight cut-off of 7000 (Membra-Cel; Viskase, Inc., Chicago, IL, USA)), which was filled with an amount according to 20 mg of formulation. Then, dialysis bag was immersed in 20 mL of phosphate buffered solution (PBS) including 0.1% (w/v) of tween 80. Aliquots of 1 mL samples were withdrawn from the medium and replaced with the same volume of fresh dissolution medium at an indicated time. The concentrations of the released drugs were determined by HPLC as described above.

### Cell culture

MCF-7/ADR cell line was purchased from the American Type Culture Collection (ATCC, Manassas, VA, USA). The cells were cultured in Dulbecco’s modified Eagle’s medium (DMEM) supplemented with 10% fetal bovine serum (FBS) and 100 units/mL penicillin in a humidified atmosphere of 5% CO_2_ at 37°C.

### Cellular uptake

To evaluate quantitative uptake of paclitaxel, the cells were seeded into a 6-well plate at the density of 1 × 10^6^ cells per well. After 24 h incubation, the cells were further incubated with different formulations at different concentrations at 37°C. After the incubation period, the cell medium containing different formulations was removed from the wells and the cells were washed with cold PBS. The cells were lysed for 10 min by addition of 1% Triton X-100 (400 μL) per each well. After that, an aliquot of the cell lysate was used to measure the total cell protein amount by the BCA assay. The concentration of paclitaxel in the cell lysate was measured by HPLC as described above.

For cellular uptake of curcumin, the cells were incubated on glass base dish (Thermo scientific, USA) and were examined by confocal laser scanning microscopy (CLSM, Model: LSM5LIVE; Carl Zeiss, Wetzlar, Germany). In glass base dish, MCF-7/ADR cells were seeded at a density of 1 × 10^6^ cells per well in 1 mL of growth medium and incubated for 24 h to allow them to attach. The cells were then treated with the different formulations in growth medium. At predetermined time point (4 h), the cells were washed three times with cold PBS. Then, cells were stained with 1 μg/mL DAPI in PBS for 3 min and washed twice with PBS. The cells were observed directly under the CLSM.

In order to determine quantitative uptake of curcumin, the cells were washed three times with cold PBS, then collected by centrifugation and re-suspended in PBS (1 mL). The mean fluorescent intensity of curcumin in the cells was measured on a BD LSRFortessa flow cytometer (Becton Dickinson, USA).

Flow cytometry analysis was adapted and four separate experiments were conducted including pretreatment of 450 mM of sucrose and 80 mM of sodium azide (NaN_3_) for 0.5 h. The effect of temperature on cellular uptake was assessed by incubation at 37 and 4°C.

### Cytotoxicity assays

To determine cytotoxicity of the various formulations, the MTT assay (Sigma-Aldrich) was conducted. Briefly, MCF-7/ADR cells were seeded in 96-well plates at a cell density of 5×10^4^ cells/mL (200 μL/well). After confluency, the cells were further incubated with the various formulations for 48 h. The culture medium was replaced by MTT solution (5 mg/mL, 200 μL/well), and the cells were incubated for a further 4 h. The supernatant was carefully removed, and 200 μL of DMSO was added to the each well. The plates were then placed in an incubator for 30 min. The absorbance values of each well were measured at 570 nm using a microplate reader (Sunrise™; Tecan, Austria).

### Western blotting

MCF-7/ADR cells were lysed for 10 min and harvested by centrifugation at 15,000 g for 10 min. Aliquots from the suspension were resolved on a 10% sodium dodecyl sulfate-polyacrylamide gel (SDS-PAGE) at 100 V for 120 min and electrotransferred onto a polyvinylidene difluoride (PVDF) membrane using a PowerPac^TM^ HC (BIO-RAD, Hercules, USA). The membrane was blocked in 5% skim milk solution at room temperature for 1 h. P-gp (170 kDa) or β-actin (42 kDa) was detected by treating the samples with p-gp antibody C219 (1: 50 dilution, Thermo scientific, Rockford, USA) or β-actin antibody (1:3000 dilution, Cell Signaling Technology, Danvers, USA) at room temperature for 2 h, followed by IgG-HRP: sc-2004 (1:2000 dilution, Cell signaling Technology, Danvers, USA) as the secondary antibody at 4°C for overnight.

### Immunofluorescence staining

In glass base dish, MCF-7/ADR (1 × 10^6^) cells were seeded on glass base dish (Thermo scientific, USA) and incubated for 24 h to allow them to attach. The cells were then treated with the different formulations in growth medium. At predetermined time point (4 h), the cells were washed three times with PBS. Then, cells were treated with p-gp antibody C219 (1:100 dilutions, Thermo scientific, Rockford, USA) for 2 h, followed by Alexa 555 antibody 1:1000 dilutions, Thermo scientific, Rockford, USA). Then, cells were stained with 1 μg/mL DAPI in PBS for 3 min and washed twice with cold PBS. Fluorescence images of the cells were observed directly using the CLSM.

### Statistical analysis

Student’s *t*-test was used to compare the groups. A *p* < 0.05 as considered to indicate statistical significance. All data are expressed as the mean ± standard deviation from three independent experiments.
